# Water strategy improves the inflorescence primordia formation of 'Arra 15' grapevine in the Brazilian semiarid region

**DOI:** 10.1038/s41598-024-57215-7

**Published:** 2024-03-20

**Authors:** Cíntia Patrícia Martins de Oliveira, Welson Lima Simões, Agnaldo Rodrigues de Melo Chaves, Jucicléia Soares da Silva, Larissa Pereira Ribeiro Teodoro, Liliane Santos de Camargos, Ricardo Gava, Yuri Rafael Alves Sobral, Paulo Eduardo Teodoro

**Affiliations:** 1 Department of Agronomy, State University of São Paulo (UNESP), Ilha Solteira, SP 15385-000 Brazil; 2 Center for Agricultural Research in the Semi-Arid Tropics, Brazilian Agricultural Research Corporation (EMBRAPA), Petrolina, PE 56302-970 Brazil; 3 Federal University of Mato Grosso do Sul (UFMS), Chapadão do Sul, MS 79560-000 Brazil; 4 State University of Bahia (UNEB), Juazeiro, BA 48904-711 Brazil

**Keywords:** Fertile buds, Irrigation management, Vine physiology, *Vitis vinifera* L., Water deficit, Plant sciences, Environmental sciences

## Abstract

Failure in irrigation management of grapevines grown in the Brazilian semiarid region can affect bud fertility. Adequate irrigation, considering both the development of bunches in the current cycle and the formation of fertile buds for subsequent cycles, can bring significant advances to viticulture. Therefore, the objective of this research was to investigate the effect of different irrigation levels during flowering on the formation of buds and potential bunches of 'Arra 15' grapevine and its relationship with metabolic processes. A field experiment was carried out in a commercial vineyard in Petrolina, Pernambuco, Brazil, during the 2021 and 2022 seasons. The experiment was designed in randomized blocks with four replications and five irrigation levels (70; 85; 100; 115 and 130% of crop evapotranspiration – ETc) during three production cycles. The variables fertile bud, vegetative bud, dead bud, potential fertility of the basal, median, and apical regions of the branches, number of potential bunches, reducing sugar, total soluble sugar, net photosynthesis, stomatal conductance, transpiration, and relative chlorophyll index were evaluated. The 115% ETc irrigation level improved the number of fertile buds and number of potential bunches. Irrigation level above 115% ETc increased gas exchange and relative chlorophyll index, while 70% ETc increased leaf sugar content. The most appropriate irrigation strategy is the application of 115% ETc during the flowering stage, for the increase of fertile buds and potential bunches of the next cycle, without influencing the vine metabolism. Total soluble sugars are a promising indicator of water deficit during flowering and as an indicator of vegetative bud formation for the next cycle.

## Introduction

One of the major challenges for irrigated viticulture in the Brazilian semi-arid region is the reduced productivity due to inconsistent bud fertility. Bud fertility is a quantitative measure of the potential to produce fruit, or an indicator of the number of bunches that will be harvested in the next season^1^. Thus, fertile bud formation has great economic relevance, since the number of bunches harvested is the major factor determining grapevine yield.

It should be noted that the management practices adopted in the vineyard should be focused not only on the development of the bunches, but also on favoring the formation of fertile buds for subsequent cycles. The position of the bud on the shoot is one of the main factors affecting bud fertility. The tendency for fertility to increase from the middle portion of the stems is due to a higher accumulation of carbohydrates in this region^2^. The low fertility of the basal buds may also be influenced by the lower light incidence on these buds^3^.

In grapevine, the formation of fertile buds is the result of the differentiation of undifferentiated primordia into reproductive primordia. The formation of inflorescence primordia is the most sensitive stage in the development of the reproductive organs in grapevines^4^. Inflorescence primordia is influenced by a set of environmental and endogenous factors, such as hormonal balance, branch vigor, ambient temperature, light intensity, water availability, mineral nutrition, and crop management^5^. Among these, water availability deserves to be highlighted, mainly because it influences the bud formation^6^ and is involved in morphological, biochemical, and physiological processes in plants^7^.

Water stress during flowering in the previous season determines most of the variations in bud fertility in the next season^6^. Cabral et al.^8^ found that 25% of ETc is an appropriate strategy for ensuring yield, quality and vegetative vigor in Touriga Franca vines grown in Mediterranean regions. Williams et al.^9^ found that irrigation with 140% of ETc increases vegetative vigor and shading in the renewal zone.

Bud fertility can be affected by both soil and climate conditions and cropping system^1^. Thus, carrying out appropriate management practices for each cultivar in each region is essential. In this regard, the hypothesis of this research is that during the inflorescence primordia formation step, the reduction of the irrigation rate can interfere in metabolic events of the vine without impairing the differentiation of buds for the next cycle.

Deepening this field of knowledge may bring advances in viticulture management, contributing to the increase of yield over several cycles. In this perspective, the objective of this study was to investigate the effect of different irrigation rates during flowering on the formation of buds and potential bunches of 'Arra 15' grapevine and its relationship with metabolic processes.

## Results

Table 1 contains the p-value of the F test for each source of variation and variables evaluated. There was a significant effect (p-value < 0.01) on irrigation rates for the variables fertile bud (FB), vegetative bud (VB), dead bud (DB), potential fertility of basal branch region (PFBB), middle branch region (PFMB), apical branch region (PFAB), number of potential bunches (NPB), reducing sugar content (RS), total soluble sugar (TSS), net photosynthesis (*A*), stomatal conductance (*g*_*s*_), and transpiration (*E*) and relative chlorophyll index (RCI). The interaction between water levels and cycle indicates that there are other climatic factors affecting these variables.

**Table 1:** P-values of the F-test from the analysis of variance for the variables fertile bud (FB), vegetative bud (VB), dead bud (DB), potential fertility of basal branch region (PFBB), middle branch region (PFMB), apical branch region (PFAB), number of potential bunches (NPB), reducing sugar content (RS), total soluble sugar (TSS), net photosynthesis (*A*), stomatal conductance (*g*_*s*_), and transpiration (*E*) and relative chlorophyll index (RCI).

SV	FB	VB	DB	PFBB	PFMB	PFAB	NPB	RS	TSS	*A*	*g*_*s*_	*E*	RCI
Blocks	27.41^ns^	0.44^ns^	2.33^ns^	13.77^ns^	17.29^ns^	57.72^ns^	693.77^ns^	378.34^ns^	42,939.51^ns^	0.28^ns^	0.00^ns^	0.06^ns^	3.22^ns^
Cycle (C)	851.47**	8.98**	194.36**	1241.28**	931.51**	1865.32**	21,956.31**	38,803.98**	396,931.86**	630.54**	0.14**	31.06**	898.06**
Irrigation Rate (R)	326.25**	56.07**	45.70**	405.58**	375.80**	473.19**	5996.00**	14,810.28**	1,091,566.06**	25.53**	0.00**	1.60**	84.30**
CxR	8.97^ns^	1.72^ns^	3.93**	18.83^ns^	18.00^ns^	17.20^ns^	349.69^ns^	632.51^ns^	64,276.11^ns^	3.55**	0.00**	0.45**	10.97**
CV (%)	4.46	13.47	13.62	5.56	3.96	8.07	4.74	7.09	4.11	4.27	7.00	6.18	2.31

*ns* not significant, **Significance at 1% by the F test.

### Percentage of buds

**Figure 1:**
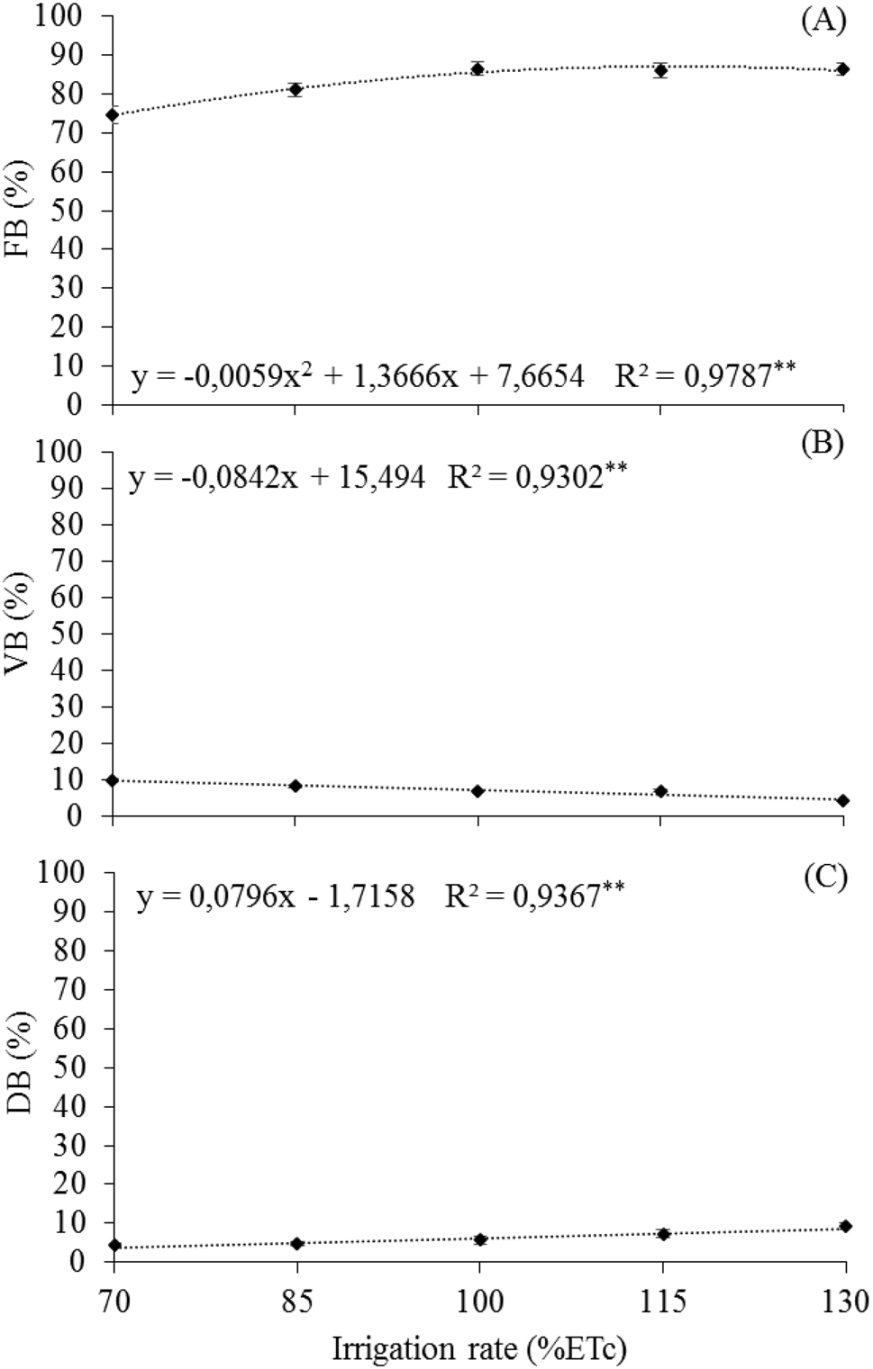
Means of the percentage of buds by presence or absence of inflorescence primordial in 'Arra15' grapevines grown under different irrigation rates in semi-arid conditions. (**A**) Fertile bud—FB. (**B**) Vegetative bud—VB. (**C**) Dead bud—DB. * and **Significant at 5% and 1% probability, respectively.

For the variables related to the process of bud differentiation by the presence or absence of inflorescence primordia, a quadratic response was observed for fertile buds (FB), with a maximum mean value of 86.8% achieved with the 115.8% ETc (Fig. 1A). For vegetative buds (VB), there was a linear response, with a higher mean value of 9.7% (Fig. 1B) and 9.1% for dead bud (DB) (Fig. 1C), reached, with the 70 and 130% ETc rates, respectively.

### Potential fertility and number of potential bunches

**Figure 2:**
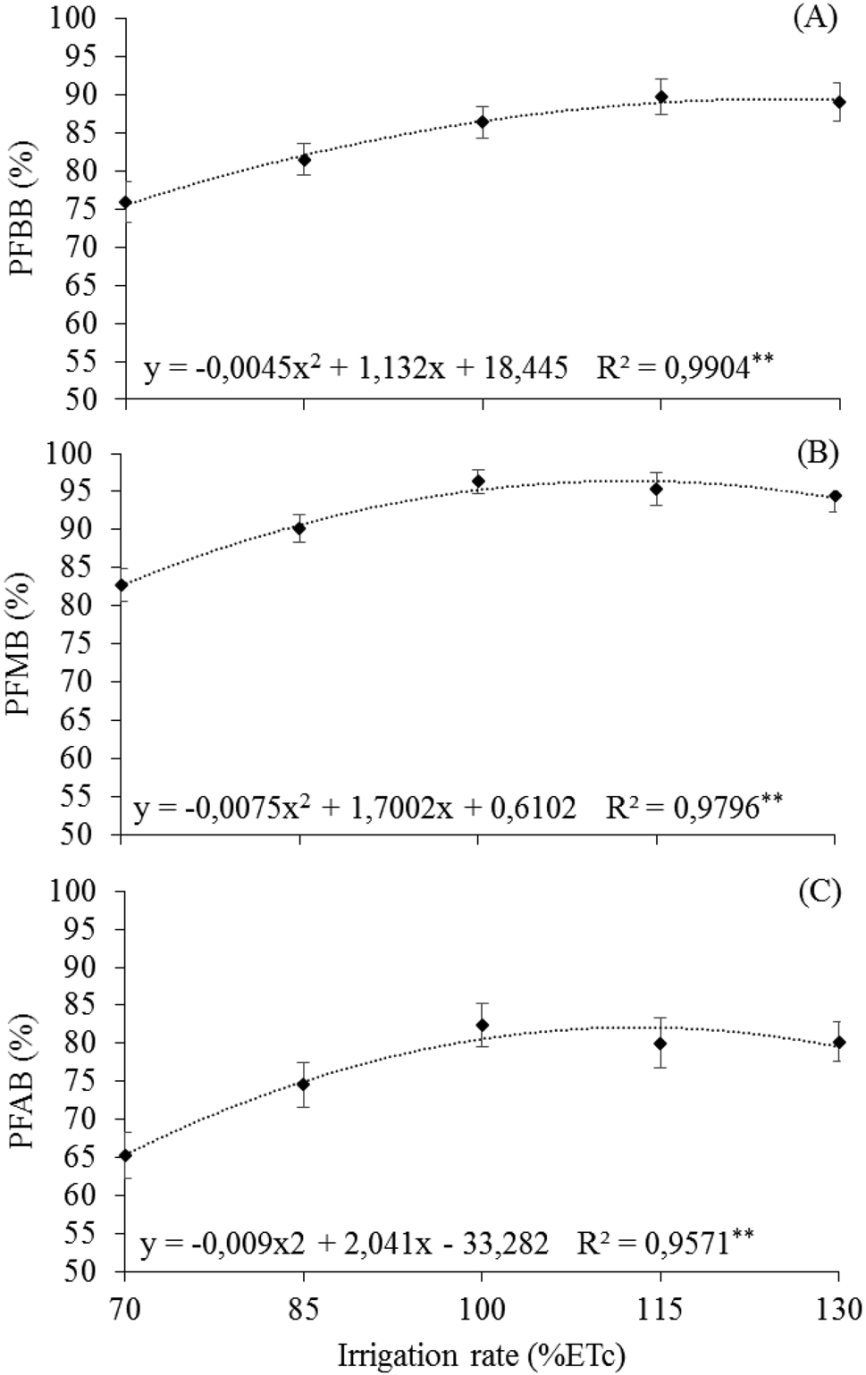
Means of potential fertility of 'Arra15' grapevines grown under different irrigation rates in semi-arid conditions. (**A**) Potential fertility of the basal branches—PFBB. (**B**) Potential fertility of the middle branches—PFMB. (**C**) Potential fertility of the apical branches—PFAB. * and **Significant at 5% and 1% probability, respectively.

For the variables related to bud potential fertility, quadratic responses were observed with maximum points of 89.64% for potential fertility of the basal branches -PFBB (Fig. 2A), 96.97% for potential fertility of the middle branches—PFMB (Fig. 2B) and 83.43% for potential fertility of the apical branches—PFAB (Fig. 2C), reached with the rates of 125.78% 113.35% and 113.39% ETc, respectively.

**Figure 3:**
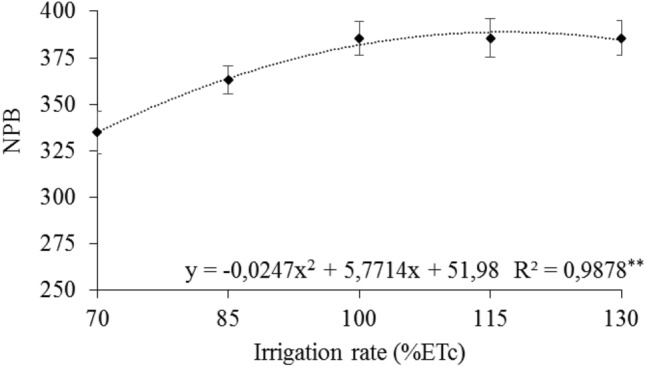
Means of number of potential bunches (NPB) of 'Arra15' grapevines grown under different irrigation rates in semi-arid conditions. * and **Significant at 5% and 1% probability, respectively.

Regarding the variable number of potential bunch (NPB), a quadratic response was observed with maximum points of 389.11 reached under condition of 116.82% ETc (Fig. 3).

### Physiological variables

**Figure 4:**
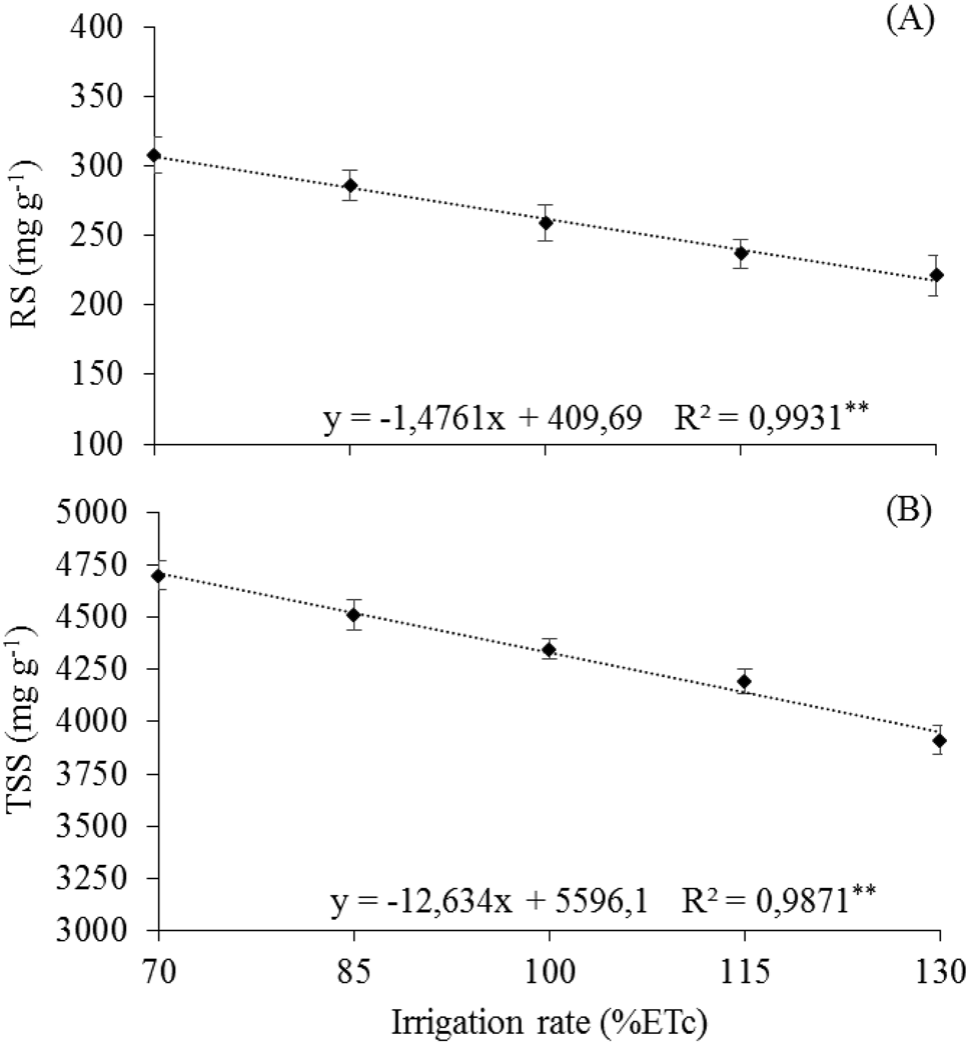
Means of leaf reducing sugars of 'Arra15' grapevines grown under different irrigation rates in semi-arid condition. (**A**) Reducing sugar content—RS. (**B**) Total soluble sugar content—TSS. * and **Significant regression at 5% and 1% probability, respectively.

In Fig. 4, the regression equation allowed estimating the effect of irrigation rates on leaf physiological variables. A decreasing linear response was observed as a function of irrigation rates, with the highest mean values of 307.08 and 4700.51 mg g^–1^ for RS and TSS, respectively, both reached with the 70% ETc rate.

**Figure 5:**
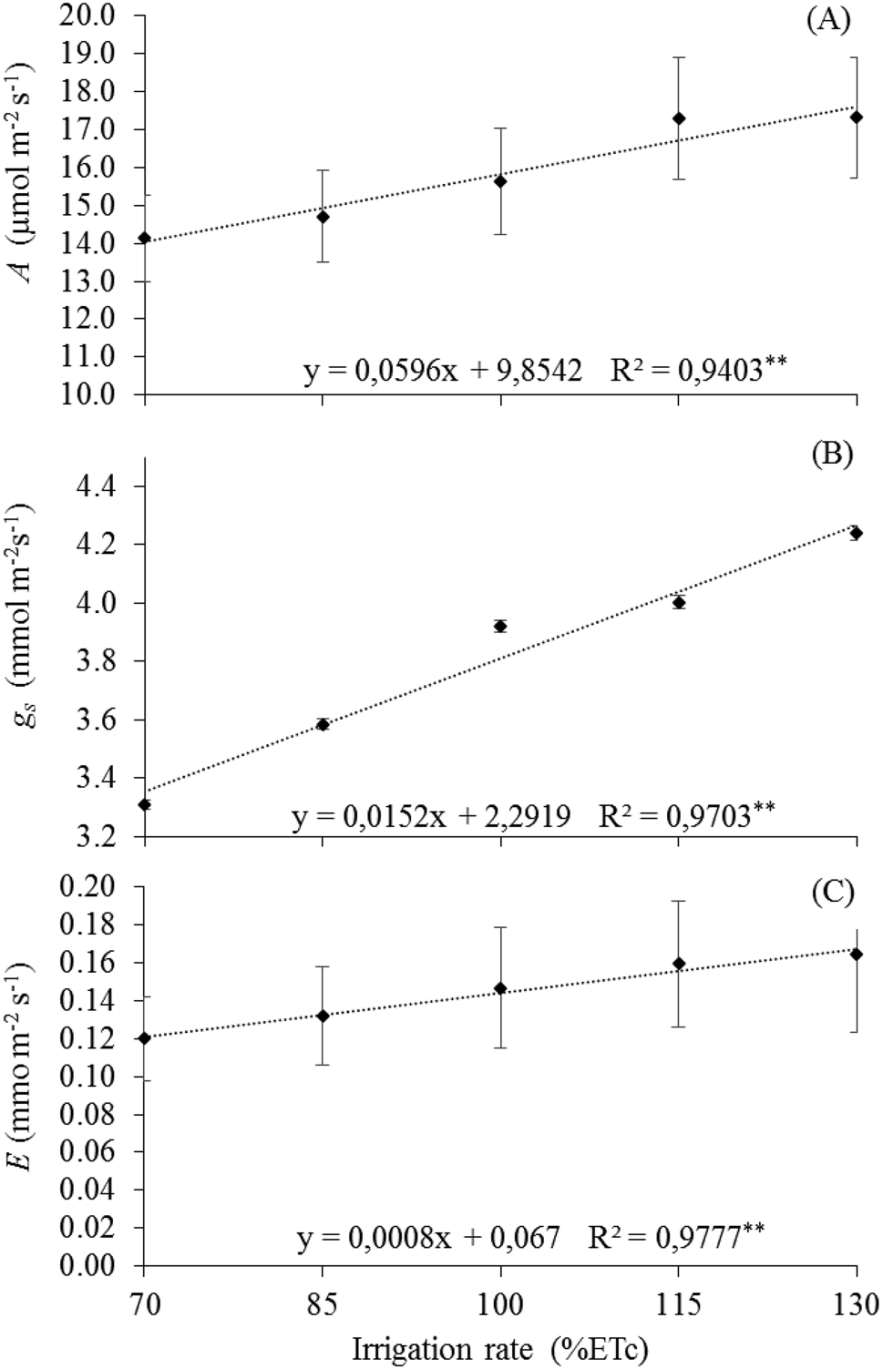
Means of leaf gas exchange in 'Arra15' grapevines grown under different irrigation rates in semi-arid conditions. (**A**) Net photosynthesis—*A*. (**B**) Stomatal conductance—*g*_*s*_. (**C**) Transpiration—*E*. * and **Significant at 5% and 1% probability, respectively.

According to the regression equations for the variables related to gas exchange, an increasing linear response was observed, with the highest mean value of 17.32 µmol m^–2^ s^–1^ for net photosynthesis (Fig. 5A), 4.24 mmol m^–2^ s^–1^ for transpiration (Fig. 5B), and 0.16 mol m^–2^ s^–1^ for stomatal conductance (Fig. 5C), respectively, achieved with 130% ETc.

**Figure 6:**
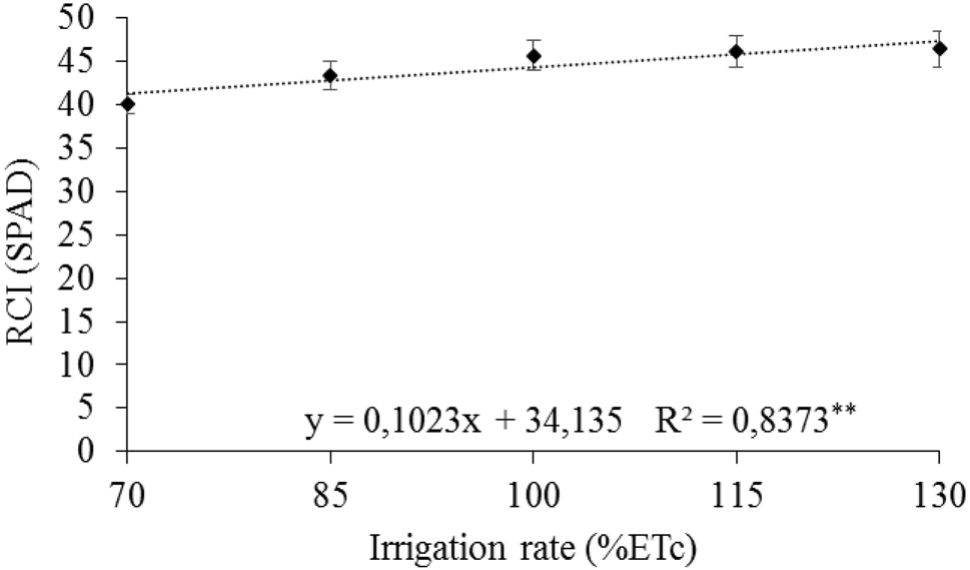
Mean relative chlorophyll index—(RCI) in leaves of 'Arra15' grapevines grown under different irrigation rates in semi-arid conditions. * and **Significant regression at 5% and 1% probability, respectively.

It can be seen in Fig. 6 that the regression equation allowed estimating the effect of irrigation rates on the relative chlorophyll index (RCI), with an increasing linear response as a function of irrigation rates, with the highest average value of 46.42 RCI unit, reached with the irrigation rate of 130% ETc.

### Canonical variables analysis

**Figure 7:**
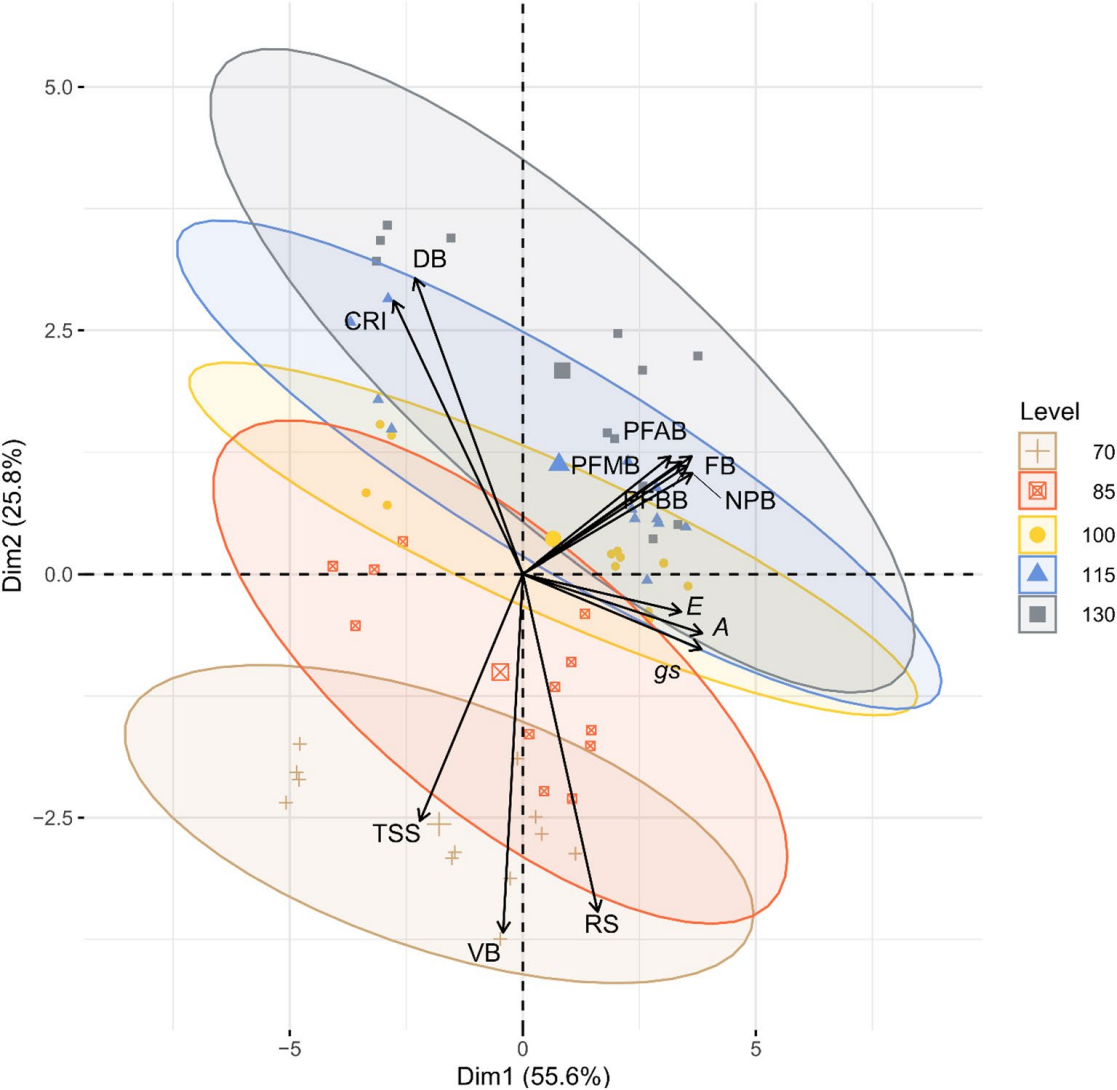
Biplot for Canonical Variables analysis in 'Arra 15' grapevine under different water regimes, studied as a function of the variables fertile buds (FB), vegetative buds (VB), dead buds (DB), potential fertility of basal branches (PFBB), potential fertility of middle branches (PFMB), potential fertility of apical branches (PFAB), number of potential bunches (NPB), reducing sugar (RS), total soluble sugar (TSS), relative chlorophyll index (CRI), net photosynthesis (*A*), stomatal conductance (*g*_*s*_), transpiration (*E*).

By canonical variables analysis (Fig. 7), the colored dots represent the irrigation rates (70; 85; 100; 115 and 130% ETc), while the arrows near and in the same direction demonstrate correlated variables (≥ ± 0.0). The first two canonical variables (DIM1 and DIM2) explained 81.4% of the total variation contained in the original dataset.

In Fig. 7, it is observed that the 115 and 130% of ETc rates are similar to each other, and positively favor the formation of FB, PFBB, PFMB, and PFAB, resulting in a higher NPB. The 115% and 130% ETc irrigation rate also exerted an influence on DB and RCI variables, which were inversely proportional to RS. The variables related to gas exchange were favored when they were close to the irrigation rate of 100% ETc. On the other hand, the variables VB and TSS were favored under 70% ETc condition.

### Pearson correlation

**Figure 8:**
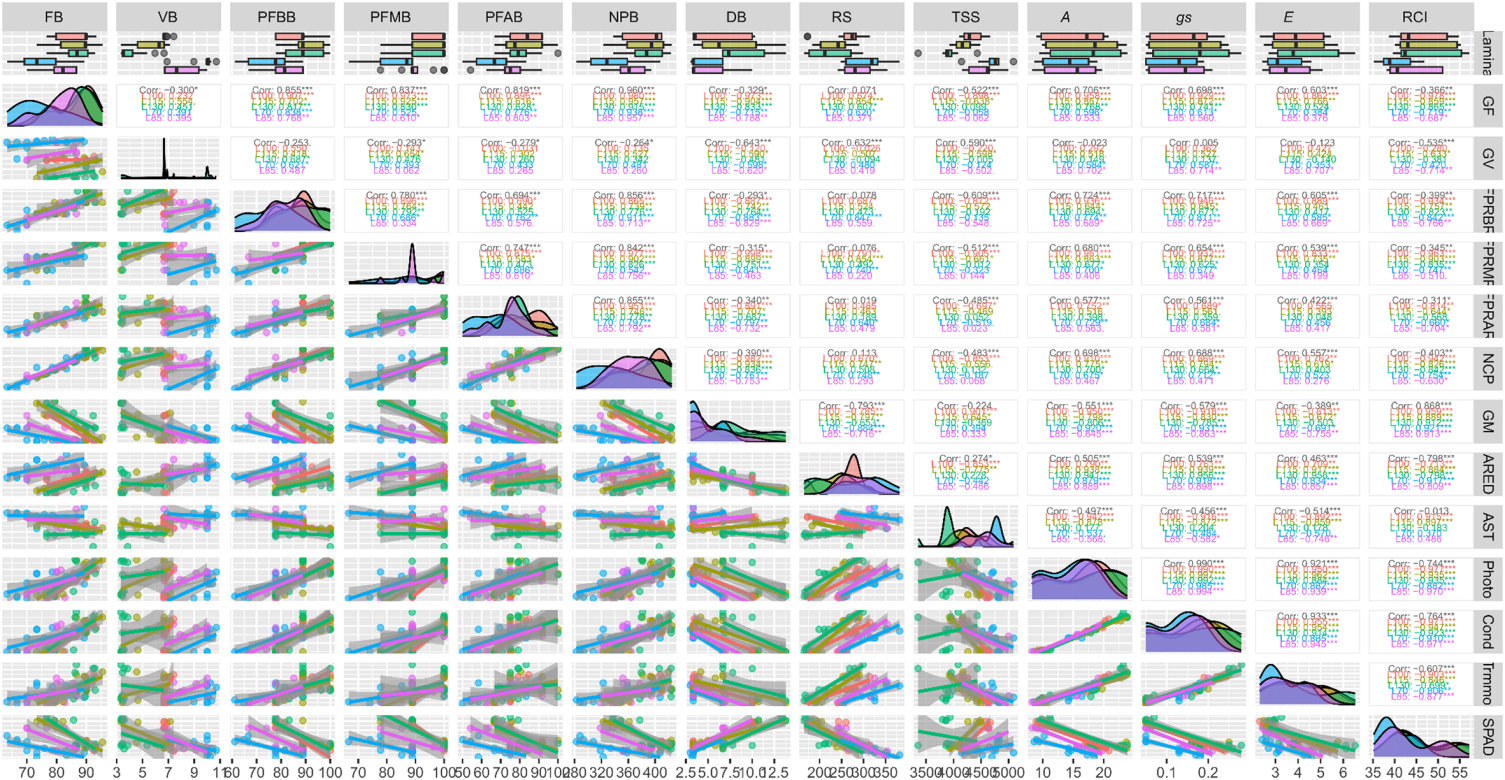
Biplot for Pearson's correlation analysis between the variables fertile bud (FB), vegetative bud (VB), dead bud (DB), potential fertility of basal branches (PFBB), middle branches (FPMB) and apical branches (PFAB), number of potential bunches (NPB), reducing sugar content (RS), total soluble sugar content (TSS), relative chlorophyll index (RCI), net photosynthesis (*A*), stomatal conductance (*g*_*s*_), and transpiration (*E*). Values shown in black indicate the overall correlation, while colored values indicate the correlation between irrigation managements. *, ** and ***Significant at 5, 1 and 0.1% probability by *t* test, respectively.

According to the Biplot presented in Fig. 8, high, positive, and significant correlations (P < 0.01) were observed between FB × NPB (0.960), *A* × *g*_*s*_ (0.990), *A* × *E* (0.921), and *g*_*s*_ × *E* (0.933). The other variables are associated with different magnitudes, highlighting the positive and significant correlations (P < 0.01) between FB x *A* (0.706) and DB × RCI (0.868).

## Discussion

However, it was expected that the treatment 130% ETc would have a greater influence on the percentage of fertile buds, considering the higher photosynthetic rate in this treatment (Fig. 8A), but this was not verified here. The decrease in sugars under 30% ETc condition may have influenced physiological disturbance, affecting the formation of inflorescence primordial and hence contributing to the incidence of DB by necrosis. It is worth noting that most of the soluble non-structural carbohydrate content of grapevine leaves consists of reducing sugars (glucose and fructose) and total soluble sugars (hexoses such as sucrose)^10^.

In grapevine, the process of differentiation of the vegetative bud into a fertile bud occurs at three well-defined stages: formation of the undifferentiated primordium, differentiation into an inflorescence primordium, and bloom formation^5^. The formation of the undifferentiated primordium and the differentiation into an inflorescence primordium occur during the growth of the branches from the previous cycle, lasting 45 to 60 days from sprouting, while bloom formation occurs shortly before and during the sprouting of the next cycle^4^.

In this regard, the results found here indicate that the irrigation rate applied at the formation of the inflorescence primordia in a cycle influences the number of fertile buds for the next cycle in 'Arra 15' grapevines. Guilpart et al.^6^ when evaluating 'Shiraz' and 'Aranel' vines grown in Mediterranean climate, found that irrigation with 60% ETc during flowering of the previous season determines most of the variations in bud fertility of the next season, supporting our findings.

During the period from 45 to 60 DAPP, the irrigation rate of 70% of ETc possibly caused an excessive reduction of water, disfavoring the process of bud differentiation, resulting in an increased percentage of vegetative buds in the following cycle. A contrasting pattern was found in 'Shiraz' and 'Aranel' grapes grown in Mediterranean climate conditions, where a mild water deficit (60% ETP) during flowering in the previous season led to an increased number of fertile buds in the subsequent season^4^.

Bud necrosis in grapevine is a physiological disorder, usually characterized by abortion of primary and secondary buds, and is associated with inadequate carbohydrate levels^11^. However, it is likely that excessive water availability may have an adverse effect on the formation of fertile buds, since the 130% ETc condition led to an increase in dead buds. Similarly, irrigation with 140% of ETc increased bud necrosis in 'Thompson Seedless' vines grown in California, as a consequence of increased vegetative vigor and shading in the renewal zone^9^.

Irrigation rates below 100% ETc, applied at the stage of inflorescence primordia formation of 'Arra 15' grapevine can reduce bud fertility in any position of fertile buds on the branch. According to Guilpart et al.^6^, water shortage during the floral differentiation process leads to decreases in bud fertility in the subsequent cycle, supporting the findings reported here.

Higher fertility in the middle branches when compared to the basal and apical region, regardless of the applied irrigation rate, may be related to genetic traits of the cultivar itself. In 'Arra 15' vines grown in a desert climate, there was also an increase in bud fertility from the basal buds to the middle buds and a gradual decrease towards the distal buds^12^.

However, the amount of water made available somehow influences the deviation of photoassimilates from branch growth to the development of inflorescence primordia^4^. Baeza et al.^3^ reported that a higher water availability in the grapevine canopy region forms a microclimate around the bud and may influence bud fertility. Meanwhile, Vilar et al.^1^ reported that the concentration of fertile buds in the more distal region of the branch denotes a higher vegetative activity.

The formation of inflorescence primordia in the buds during the previous cycle defines the potential number of bunches that the vine will produce in the subsequent cycle^1^. In this scenario, it is evident that the use of water supply from 100% ETc during the inflorescence primordia formation has a greater influence on the potential number of bunches per plant for the next crop cycle. This information is of considerable interest to the winegrower, as it allows him to estimate the production and pruning management for the next cycle.

The determination of the number of potential bunches is affected by water availability during the initial stages of vegetative bud differentiation into fertile ones from the previous cycle^6^. Thus, water supply above 100% ETc may be an appropriate strategy for the bud differentiation process in 'Arra15' grapevine, favoring the number of potential bunches per plant for the following cycle.

According to the canonical variables, the 115 and 130% ETc levels had a stronger influence on the bud differentiation process, since they favored the formation of FB, PFBB, PFMB, PFAB, and consequently the NPB. This means that maintaining high irrigation levels between 45 and 50 DAPP positively influences bud fertility in 'Arra 15' grapevines. According to Balint and Reynolds^13^, to achieve high bud fertility and productive potential for the next cycle, the vine needs high water demand during the differentiation of the inflorescence primordia, supporting the findings of this study.

The gradual reduction of the irrigation rate during the stage of inflorescence primordia formation, across all three crop cycles, causes an increase in RS and SST levels in 'Arra15' leaves. These results are similar to those obtained in Malbec vines grown in arid climates, where the lowest level of irrigation (25% ETo) showed a higher content of total soluble sugars than the vines under the highest level of irrigation (100% ETo)^14^.

Increasing sugars under irrigation level of 70% ETc is likely due to the reduction of translocation of these photoassimilates to the drains. Accumulation of free sugars in the leaves may be due to the disturbances caused by stressful conditions such as the reduction of water content in leaf tissues, reducing the translocation of sugars to other organs^15^.

Fahim et al.^16^ reported the synthesis of soluble sugars in the cytosol is probably prioritized to maintain the plant's metabolic activity, even when water deficit reduces the rate of CO_2_ assimilation. Meanwhile, Oliveira et al.^15^ reported that the accumulation of free sugars in the leaves with decreasing water content in the soil maintains cell turgor contributing to plant functioning. However, in order to confirm osmotic adjustment, assessing other physiological parameters that were not investigated in these studies is necessary.

By canonical variables analysis, VB and TSS were more sensitive to a reduced irrigation level when compared to the other variables. Possibly, buds under 70% ETc condition receive less carbohydrates from photosynthetically active leaves, negatively affecting the differentiation process of meristematic tissues in inflorescence primordia. Water status influences the formation of inflorescence primordia, either directly through the water volume available for the biosynthetic processes that occur during cell division and growth, or indirectly through its effect on photosynthetic activity, mineral nutrition, and hormonal balance^7^.

According to Monteiro et al.^5^, several endogenous and environmental factors influence the differentiation of meristematic tissue, where the vegetative bud instead of developing into inflorescence primordia, develops into tendril primordia, or sprout primordia. Thus, the irrigation rate application with 70% of ETc associated with an increase in leaf total soluble sugars between 45 and 50 DAPP is an indication of greater VB formation for the next cycle. The determination of TSS as an indicator of VB is advantageous because it is easier to measure, faster, labor-saving, and provides results regarding disturbances in the bud differentiation process.

The positive and strong correlation between FB and NPB was already expected, since bud fertility is a quantitative measure of the vine's potential to produce fruit or an indicator of the number of bunches that will be harvested in the next season^1^.

The decreases in *A*, *E* and *g*_*s*_ with reduced irrigation are associated with stomatal closure and limitations in CO_2_ assimilation and transpiration. A similar result was found in 'Cabernet Sauvignon' grapevine grown in continental climate in USA^2^, on 'Chardonnay' grapevine grown in a temperate climate in Canada^13^ and in 'Syrah' grapevine grown in a semi-arid climate Brazil^17^.

Under drought condition, guard cells reduce stomatal opening, decreasing transpiration to avoid leaf dehydration, which limits CO_2_ diffusion into the leaf mesophyll and reduces photosynthesis^7^. Vines under water deficit tend to close their stomata to reduce canopy water loss, which prevents sharp declines in plant water potential and saves water for use during the growing season^18^. In this regard, the 'Arra 15' vine is sensitive to water deficit, as it induces the closure of stomata, causing reductions in transpiration and photosynthesis. Thus, the application of the irrigation level at 70% ETc during the period from 45 to 50 DAPP can reduce gas exchange, providing a decrease in the production of photoassimilates.

Each grapevine cultivar has different stomatal response to a given soil water potential^19^. Thus, the greater influence of the 100% ETc level on the variables A, E, g_s_, indicates that the soil water status provided by this level allows a higher capacity to keep the leaf active.

The strong and positive correlations between A, E and g_s_ is also expected, as variations in gas exchange occur as a function of stomatal movement. Keller et al. (2016) found a strong and positive correlation between irrigation rates and A, E, and g_s_ in Cabernet Sauvignon vines grown in a continental climate in the Columbia Valley, Washington, USA, corroborating our findings. Sequestering nutrients from photosynthesis for developing reproductive organs is common in some grapevine cultivars^10^. Thus, the correlation between FB and A indicates that the higher the production of photoassimilates from photosynthesis, the higher the formation of fertile buds.

Reductions in chlorophyll levels may also represent an acclimation response to water restriction, as the plant tends to conserve energy and hence capture less light energy, possibly avoiding photooxidative stress^15^. Thus, given the relationship between energy absorption and transfer, the levels of photosynthetic pigments may be related to net photosynthesis as well as to the growth and adaptation of 'Arra 15' to stress condition.

The decrease in photosynthetic pigment content due to water deficit (25% of field capacity) was diagnosed in Iranian native grape cultivars^16^. This authors reported that the loss of photosynthetic pigment under water deficit conditions occurs due to damage to the enzymes associated with photosynthesis, supporting the results obtained here.

The positive correlation between RCI and DB suggests that the increase in RCI may indicate disturbances in the bud differentiation process. It is likely that the intensity of this correlation is favored in conditions of excess soil water, which is supported by the canonical analysis, which showed an influence of the 130% ETc irrigation level on DB and RCI. Excessive irrigation increases the necrosis of grapevine buds, as a result of a higher vegetative vigor and shading in the renewal zone^9^.

The "b" chlorophyll content tends to increase in environments with a higher level of shading to enable the capture of energy in other wavelengths and transfer it to the "a" chlorophyll, which is responsible for photochemical reactions of photosynthesis^20^. It is possible that the increased RCI reflects an increase in "b" chlorophylls, and may indicate disturbances in the bud differentiation process. Thus, through RCI measurements taken between 45 and 50 DAPP, it is possible to adjust irrigation level to reduce the percentage of dead buds for the next cycle.

In light of the above, the water supply and its relationship with the physiology and morphology of reproductive organs may contribute to the decision-making by winegrowers. The findings reported in this research help to improve irrigation management from one cycle to another. Further studies should be focused on the processes of inflorescence primordium formation and how and when its metabolic pathways are affected, as it is a process greatly influenced by the water status of the vine.

The most appropriate irrigation strategy is the application of 115% ETc during 45 to 50 DAPP for the increase of fertile buds and potential bunches of the next cycle. The 115% ETc irrigation level does not impair the metabolism of 'Arra 15' grapevine grown in semi-arid condition. The highest percentage of fertile buds in 'Arra 15' is found in the middle region of the branch. TSS can be used as an indicator of water deficit during flowering and as an indicator of VB formation in the next cycle.

## Material and methods

### Experimental design and plant material

Field experiment was carried out in a commercial vineyard in the Senador Nilo Coelho Irrigated Project, located in the municipality of Petrolina, Pernambuco, Brazil (09° 22′ 26.75" S and 40° 38′ 4.29" O), during the years 2021 and 2022, period corresponding to three production cycles. Each production cycle began with production pruning. In the first cycle, production pruning was carried out on August 9, 2021; in the second cycle, production pruning was carried out on January 6, 2022; and in the third cycle, production pruning was carried out on August 15, 2022.

The vines assessed were nine-year-old 'Arra15', propagated by grafting (rootstock '1103 Paulson'), with a spacing between plants of 2.5 × 4.0 m, conducted in pergola system. The experimental research followed relevant institutional, national and international guidelines and legislation.

The American cultivar 'Arra 15' or 'ARRAFIFTEEN' was developed by the Grapa Company together with the Guimarra Vineyards Corporation in California, USA^21^. It is a white seedless grape that has high natural fertility of the buds, with two bunches per shoot on average, resistance to cracking of the berries due to rain during the ripening period, a low rate of degranulation, and a high yield with around 40 to 50 bunches per vine^21^.

**Figure 9:**
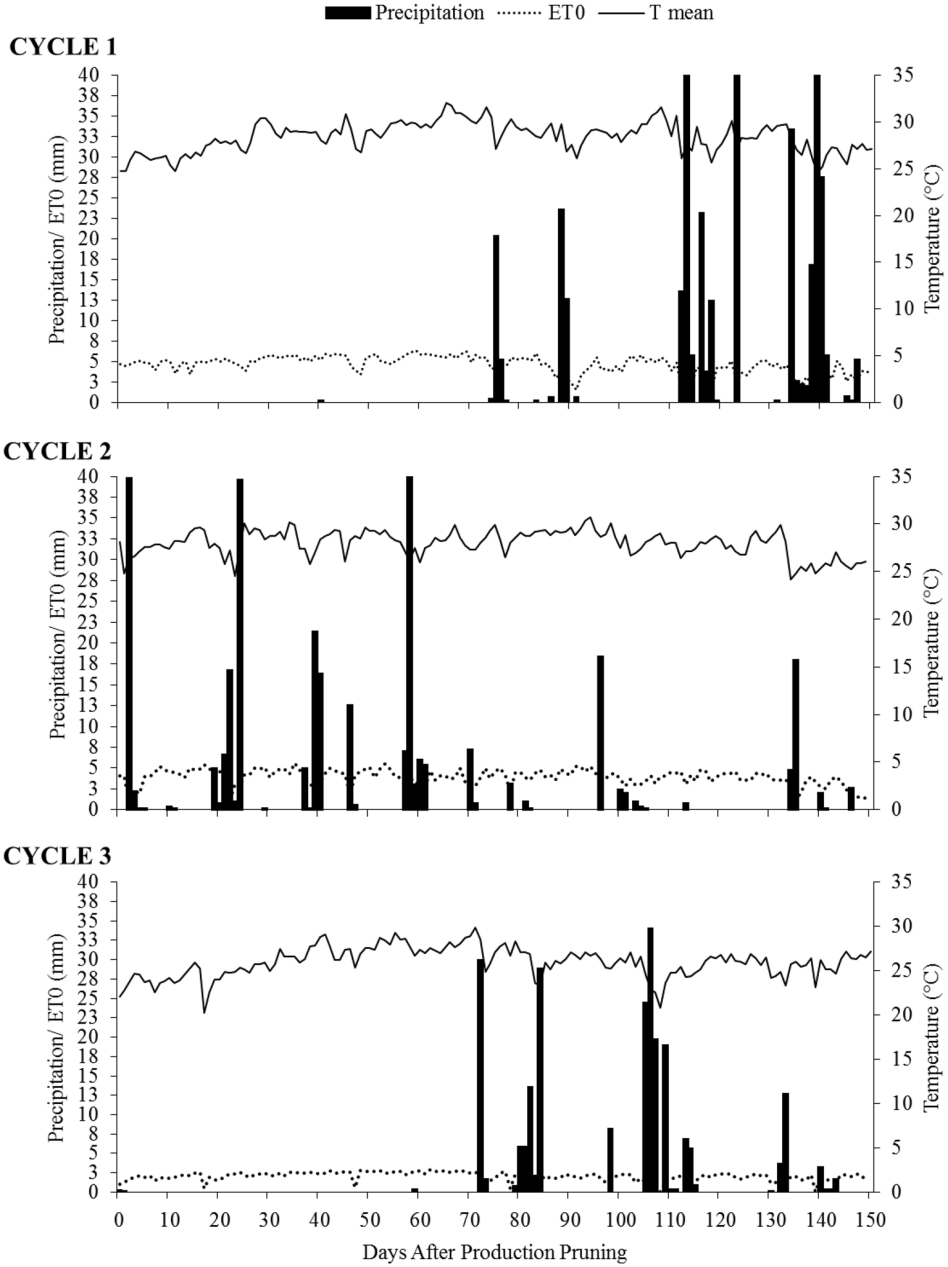
Seasonal variations of mean temperature (T mean), reference evapotranspiration (ET0), and precipitation of three consecutive crop cycles of 'Arra 15' grapevine. *DAPP* days after production pruning. Production pruning of cycle 1 carried out on 08/09/2021, cycle 2 on 01/06/2022 and cycle 3 on 08/15/2022.

The climate of the region according to the Köeppen classification is BSh type, which means semi-arid region, very warm and with irregular rainy season. The region has annual rainfall averages of approximately 500 mm, irregularly distributed and concentrated from November to April. It has an average annual relative humidity of 66% and an average annual air temperature of 26.5 °C, with the highest peaks from October onwards^22^. The average monthly values of maximum and minimum air temperature (°C) and precipitation (mm) over the three study cycles were obtained from the Automatic Agricultural Weather Station installed near the experiment site, and are shown in Fig. 9.

Irrigation system consisted of two drip lines per planting row, installed at 2 m above the ground, with drippers spaced by 0.4 m. The irrigation shift was daily with a flow rate of 2.0 L h^–1^. Crop management, phytosanitary control, management of fertilization and vegetation were carried out by the farm according to the recommendations for viticulture in the São Francisco Valley^1^. Soil analysis were performed before implementation/beginning of the experiment at depths 0–30 cm and 30–60 cm (Table 2).

**Table 2:** Chemical analysis of sandy loam soil from the experimental area of 'Arra 15' grapevine at the depths (Prof.) of 0–30 cm and 30–60 cm.

Depth (cm)	Chemical attributes (cmolc/dm^–3^)							
	Ca	Mg	Na	K	SB	H + Al	T	Al^+++^
0–30	4.9	3.2	0.10	0.69	8.85	0.00	8.85	0.00
30–60	3.7	1.4	0.05	0.26	4.45	0.00	4.45	0.00

Depth (cm)	pH	%	gkg^-1^		mgdm^–3^			
	1:2.5	V	C	OM	P			
0–30	7.4	100	24.3	42.0	438.27			
30–60	7.2	100	8.5	14.6	246.16			

The experimental design used was randomized blocks with four replications, and the treatments consisted of five irrigation rates (70; 85; 100; 115 and 130% of crop evapotranspiration, —ETc) analyzed in three crop cycles. The experiment comprised a total of 20 plots, where each experimental plot consisted of 5 plants, with the three central plants being evaluated as the experimental units, totaling 60 useful plants.

The factor irrigation rates was quantified using the equation: ETc = ET0 × Kl × Kc. Reference Evapotranspiration (ET0) was calculated by the Penman–Monteith method^23^, using data collected by a weather station installed near the experimental area. The location effect (Kl) was determined according to Albuquerque^24^, using a value of Kl = 1 when the shaded area in the vineyard reached 75%. The value of 100% ETc corresponded to the Kc already determined for seedless grapevine cultivars in the Submédio São Francisco region^25^. The Kc was then adjusted for the other treatments.

The values of the Crop Coefficient (Kc) applied to the flowering and pea-size berries (30 to 50 days after pruning—DAPP) were: 0.25 (70% of ETc); 0.40 (85% of ETc); 0.55 (100% of ETc); 0.85 (115% of ETc); and 1.1 (130% of ETc).

### Variables assessed

At 50 Days After Production Pruning (DAPP), between 9 a.m. and 10 a.m., gas exchange and relative chlorophyll index assessments, and leaf collection for biochemical analysis were performed in three useful plants of the plots. All assessments were performed on mature and fully expanded leaves exposed to the sun and located in the middle third of the plants. It is worth noting that 50 DAPP corresponds to the stage of undifferentiated primordium formation, which occurs during the growth of branches from the previous cycle, lasting 45 to 60 days after pruning^26^.

The parameters related to gas exchange were: net photosynthesis (A), stomatal conductance (g_s_), and transpiration (E), obtained through a portable infrared gas analyzer—IRGA (Model LI-6400XT, Li-Cor, Lincoln, NE, USA). The relative chlorophyll index (RCI) was obtained using a portable chlorophyll meter (Falker, model: ChlorofiLOG). The biochemical variables evaluated included the leaf contents of reducing sugars (RS), quantified by the dinitrosalicylic acid method^27^, and total soluble sugars (TSS), quantified by the antrona method^28^.

**Figure 10:**
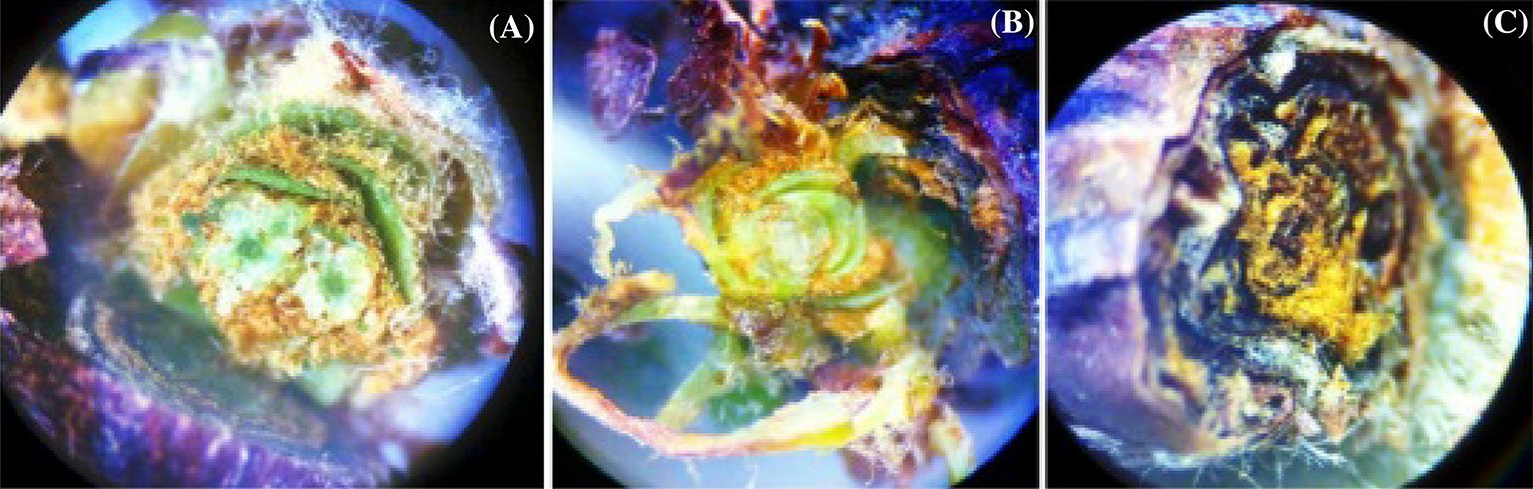
Visualization of 'Arra15' grapevine buds via cross-sections. (**A**) Fertile bud—FB, healthy bud with two inflorescence primordia, which are fragments of rounded shapes surrounding a central axis. (**B**) Vegetative bud—VB, healthy bud without inflorescence primordia. (**C**) Dead bud—DB, total loss of bud by necrosis. 30 × zoom.

At 140 DAPP or 30 days after harvest, one branch per useful plant was collected from each plot, each containing ten buds, totaling 120 buds per treatment for bud analysis. 140 DAPP corresponded to the period when the inflorescence primordium is fully developed, taking the form of an axis with numerous protuberances similar in appearance to a bunch of grapes, and these protuberances correspond to the future flowers to be formed^26^. The bud analysis was carried out according to the methodology proposed by^11^, where the buds were dissected and then classified as fertile (FB), vegetative (VB), and dead (DB) according to the presence or absence of inflorescence primordia using a stereo microscope (binocular magnifier) with 30 × zoom (Fig. 10).

For potential fertility, the position of the FB along the branch was identified in basal buds (1st to 3rd), middle buds (4th to 6th), and apical buds (7th to 10th), and the potential fertility of the basal branch region (PFBB), middle branch region (PFMB), and apical branch region (PFAB) were obtained. At the end of the evaluation period, the number of potential bunches per plant (NPB) was estimated at 50 branches per plant with ten buds per branch using the equation: $\frac{\mathrm{number \,\,of\,\, branches }\times \mathrm{number \,\,of \,\,buds }\times \mathrm{accumulated\,\, fertility}}{100}$.

### Statistical analyses

Data were submitted to analysis of variance by the F test. The effect of irrigation rate was considered fixed and the effect of cycle was considered random. In cases of significance, regression analysis was performed, testing linear and quadratic models for irrigation rates. In all cases, a 5% significance level was adopted.

Subsequently, canonical variables analysis was performed with the "candisc" package^29^ to verify the interrelationship between the treatments and variables evaluated. Finally, Pearson correlations were performed between the evaluated traits, where positive correlations were presented in green, while negative correlations were presented in red scale. The "qgraph" package^30^ was used to express the results graphically. All statistical analyses were performed using the R software^31^.

## Data Availability

The datasets used and/or analyzed during the current study available from the corresponding author on reasonable request^32^.
